# Infection pattern and negative effects of a facultative endosymbiont on its insect host are environment-dependent

**DOI:** 10.1038/s41598-019-40607-5

**Published:** 2019-03-08

**Authors:** Xiang-Dong Liu, Hai-Xia Lei, Fang-Fang Chen

**Affiliations:** Department of Entomology, Nanjing Agricultural University, Nanjing, 210095 China

## Abstract

*Regiella insecticola* is a bacterial endosymbiont in insects that exhibits a negative effect on the fitness of hosts. Thus, it is not clear why this costly endosymbiont can persist in host populations. Here, we tested a hypothesis that the infection pattern and negative roles of the endosymbiont were not constant but environmentally dependent. The grain aphids *Sitobion avenae*, belonging to different genotypes and infected with *Regiella* or not, were used in this study. We found that *S*. *avenae* populations were infected with *Regiella*, *Hamiltonella defensa*, *Serratia symbiotica* and *Rickettsia*. The predominant endosymbionts in the aphid populations varied with season. *Serratia* and *Rickettsia* were predominant from December to February while *Regiella* predominated from March to May. The vertical transmission of *Regiella* was poorer at high temperature, but following conditioning for seven generations, the transmission rate improved. *Regiella* inhibited the production of winged aphids at 25 °C, but it did not affect winged morph production at the higher temperatures of 28 °C and 31 °C. *Regiella* infection decreased the intrinsic rate of increase (*r*_*m*_) of aphids at 25 °C and 28 °C. However, at 31 °C, the effect of *Regiella* on the *r*_*m*_ varied depending on the aphid genotype and density. Thus, the negative effects of this endosymbiont on its host were environmentally dependent.

## Introduction

Insects house a wide range of facultative bacterial endosymbionts^1–4^. Facultative endosymbionts are usually connected to the fitness of their hosts, and these bacteria play beneficial, neutral or detrimental roles in shaping the biological and ecological traits of hosts^5–8^. The benefits of endosymbionts on their hosts are common^9–14^, and these benefits support symbionts that consistently colonize their hosts. Facultative bacterial endosymbionts with detrimental effects on hosts are also found in insect hosts, such as parasitoids and aphids^15–19^. However, the negative effects of these symbionts on the performances of their hosts may be compensated by their benefits. For example, *Hamiltonella defensa* significantly reduced the lifespan and reproduction of *Aphis fabae* in an environment without parasitoids *Lysiphlebus fabarum*, but it conferred strong protection to aphids against this parasitoid^20^. Some species of endosymbionts, such as *Hamiltonella* and *Serratia*, lead to decreased body weight, longevity or fecundity of their hosts^16–19^, but they increase the host resistance to natural enemies or heat stress^5,12,21,22^. Moreover, the effects of endosymbionts on hosts vary with environmental conditions^21–24^. *Rickettsia* and *Serratia* reduced the fecundities of hosts at 20 °C but increased the fecundity of hosts at 25 °C^21^. Similarly, *Hamiltonella* conferred resistance to the parasitoid *Aphidius ervi* in pea aphids under cool conditions but not at higher temperatures^23,24^. The costs or benefits of an endosymbiont on its hosts might be environmentally dependent.

Aphids are infected with many species of bacterial endosymbionts^1,25,26^, and endosymbionts can modify their life-history traits^27^. Migration and dispersal are important biological phenomena in the life history of insects^28^. Aphids exhibit wing dimorphism and can produce wingless and winged phenotypes that determine flight capacity. Environmental cues, population density, host plant quality, abiotic factors and maternal effects affect the production of winged versus wingless morphs in aphids^29^. In addition, it has been found that infection with some endosymbionts also affects the winged morph production of aphids. *Acyrthosiphon pisum* infected with the bacterial endosymbionts *Serratia* and *Rickettsia* produced more alatae than the uninfected aphids^21^. However, *A*. *pisum* containing *Regiella* produced only half the number of winged offspring under crowding conditions, compared with those aphids lacking *Regiella*^27^. Aphids harbouring different endosymbionts may have different abilities to produce alatae and to a certain extent, the endosymbiont infection determines the population dynamics: to migrate or settle. Therefore, endosymbiont infection may affect the spatial distribution of aphid populations.

To explore the roles of an endosymbiont in host insects, we usually compare target traits between symbiont-infected and symbiont-cured host lines that have identical genetic backgrounds. Therefore, feeding or injection of antibiotics to cure the target endosymbiont is an effective method and has been commonly adopted by many studies^7,16,30–33^. In previous studies, antibiotic treatment was only carried out for the aphids containing the target endosymbiont but not for the naturally uninfected aphids^7,31,32^. Therefore, the effects of antibiotics and the role of some unknown bacterial symbionts cured by the antibiotics were ignored. Therefore, in this study, we treated both the symbiont-infected and symbiont-free aphid strains using antibiotics to evaluate the effects of an endosymbiont on the life-history traits of aphids. This method is better for exploring the roles of endosymbionts in hosts.

The grain aphid, *Sitobion avenae*, is a serious pest in wheat fields. This pest is capable of long-distance migration^34–40^. In late spring, when the winter wheat matures, grain aphids generate winged morphs and migrate towards the northern regions of China where spring-wheat is grown; in autumn, the aphids migrate back to the southern regions of China where winter-wheat is grown^38–40^. Some grain aphids are infected with *Regiella*^7,41,42^, and *Regiella* infection decreases the intrinsic rate of aphid population increases^8^. This finding suggests that infection with *Regiella* has fitness costs for the grain aphid. However, the effect of *Regiella* on the production of winged/wingless morphs of this migratory aphid is still not clear. Moreover, it is unknown why the endosymbiont *Regiella* can persist in natural populations of the grain aphid even though it has a negative effect on the intrinsic rate of increase of aphids. As with other endosymbionts, protection against natural enemies may allow the symbiont to persist despite the costs associated with infection^11^. In this study, we hypothesized that endosymbionts depend on environmental factors to modulate the performance of aphid hosts. Therefore, we investigated the infection status of all known endosymbiont species in the grain aphid populations during the winter and spring of 2014–2016 in Nanjing, China. Furthermore, we examined the effects of *Regiella* on the intrinsic rate of increase and the winged morph frequency of aphid populations belonging to three genotypes under different densities and temperatures. The results reveal the effects of an endosymbiont on insect hosts under different environmental conditions.

## Material and Methods

### Aphids sampling, identification and rearing

**Figure 1:**
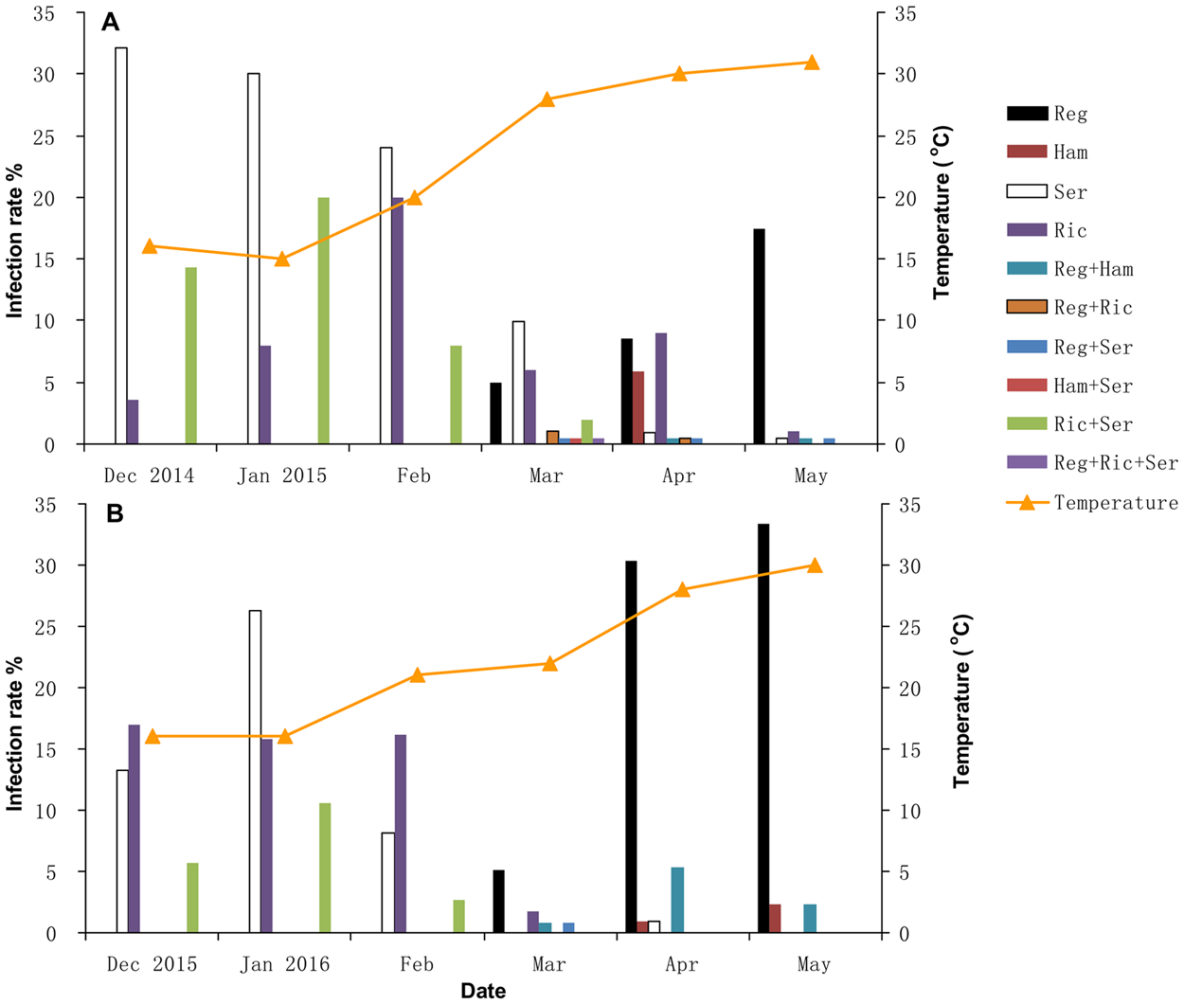
Infection rate (%) of facultative endosymbionts in the *S*. *avenae* populations of Nanjing, China from December to May during 2014–2015 (**A**) and 2015–2016 (**B**). Reg: *Regiella*, Ham: *Hamiltonella*, Ser: *Serratia* and Ric: *Rickettsia*.

The grain aphids *S*. *avenae* were collected from wheat fields in Nanjing, China during 2014–2016. Individual aphids were collected on wheat growing at least 20 m apart to reduce the risk of sampling offspring from the same mother. Each collected individual aphid was reared in Petri dishes (diameter 90 mm, height 18 mm) with 5 wheat seedlings (variety Zhengmai 5) at 20 °C, L: D = 16:8 h and RH = 55–65%. The aphid population sizes were lower in the winter and increased in the spring. The number of aphid samples was dependent on the population density in the fields. More samples were collected when the population density was higher. A total of 28, 50, 25, 201, 221 and 195 aphids were collected in December of 2014, and January, February, March, April and May of 2015, respectively. A further 53, 38, 37, 117, 112, and 42 aphids were collected in December of 2015 and January, February, March, April and May of 2016, respectively. These aphids were identified by their morphological characteristics and the COI and COII gene sequences^43,44^. The sequence identities of COI and COII were 99% and 98% with the genes of *S*. *avenae* with the GenBank submissions HM160152 and U41116, respectively. We confirmed that these aphids were *S*. *avenae*. The maximum temperature data from December to May 2014–2016 in Nanjing were collected from the website http://tianqi.2345.com/wea_history/58238.htm. In 2015 and 2016, the maximum temperature in April and May was 28–31 °C (Fig. 1); so, we studied the effects of temperatures of 25, 28 and 31 °C on endosymbionts and aphids.

### Infection frequency of facultative endosymbionts

All aphid samples collected on different dates as described above were used to examine the infection status of eight known species of facultative endosymbionts: *R*. *insecticola*, *H*. *defensa*, *S*. *symbiotica*, *Rickettsia*, *Rickettsiella*, *Spiroplasma*, *Wolbachia* and X-type using the diagnostic PCR method based on the specific primers of 16*S rRNA* or *wsp* gene of endosymbionts (Table S1). The presence of *Buchnera aphidicola* was used to determine the validity of the DNA samples from each aphid. The 16*S rRNA* gene of *Regiella* was cloned and sequenced to confirm that this endosymbiont was *R*. *insecticola*. The sequence identity was 98% with the gene sequences of *R*. *insecticola* in GenBank (JX533649 or HM156642). The infection frequency of each species of endosymbiont in an aphid population was examined based on the diagnostic PCR results.

### Establishment of *Regiella*-infected, *Regiella*-free and *Regiella*-cured aphid lines

Thirty aphid clones infected singly with *R*. *insecticola* were reared on wheat seedlings in Petri dishes as the *Regiella*-infected strains, and the other thirty aphid clones naturally lacking any known facultative endosymbionts were reared as *Regiella*-free strains. Previous studies suggested that the effects of endosymbionts on host fitness might be dependent on host and symbiont genotype or an interaction between the two^45,46^. Therefore, the genotype of each aphid clone used in this study was examined using PCR based on six microsatellite loci, S49, Sm10, Sm12, Sav1, Sav2 and Sav4^47,48^. Three genotypes were identified from these *Regiella*-infected strains and five from the *Regiella*-free strains, and the genotypes from the *Regiella*-infected and *Regiella*-free strains were different. We used the three genotypes from the *Regiella*-infected strains to establish the R1, R2 and R3 lines and chose three genotypes with different allele sizes at the microsatellite locus S49 from the *Regiella*-free strains to establish the N1, N2 and N3 lines (Table S2). All six aphid lines were reared on wheat seedlings in Petri dishes at 20 °C, a photoperiod of L:D = 16:8 h and 55–65% RH. After six generations, the infection status of the *Regiella* in all six lines was examined again, and aphids from R1, R2, R3, N1, N2, N3 lines were chosen and reared using artificial diets with antibiotics (200 µg ml^−1^ ampicillin, 100 µg ml^−1^ cefotaxime and 200 µg ml^−1^ gentamicin) to eliminate *Regiella* and estimate the influence of antibiotics on aphids^30,32^. *Regiella* was eliminated from aphids reared on antibiotic diets for two generations, and then these aphids were reared on new wheat seedlings without antibiotics. The presence of *Regiella* was checked every three generations to ensure the elimination of *Regiella*. Therefore, six symbiont-cured lines from *Regiella*-infected and uninfected strains were established and named R1-C, R2-C, R3-C, N1-C, N2-C and N3-C (Table S2).

### Transmission of *Regiella* under different temperatures

The transmission rate of *Regiella* from mother aphids to offspring was examined using the R1 and R2 lines. For each aphid line, 10 wingless adults whose mothers were infected with *Regiella* were simply selected randomly and reared in a Petri dish using wheat seedlings. After 24 h, 10 newborn offspring were maintained in a dish covered with plastic film, and all the adults and other offspring were removed. Then, the offspring were reared at a constant temperature of 20, 25 or 28 °C. When these aphids grew to adults and produced offspring, three wingless adults per dish were sampled to examine the *Regiella* infection status using PCR methodology, and the offspring from mothers with *Regiella* were reared under the same conditions for the next generation. These aphids were reared for seven generations under these conditions, and the *Regiella* infection rates were examined every two generations. There were 20 dishes per temperature, and these were kept separately throughout the process. For each generational assessment of *Regiella*, 3 out of 10 aphids in a dish were chosen, and a total of 30 aphids from 10 dishes were considered a block when computing the infection rate. Two blocks (60 aphids) were used for each generation.

### Fitness of *S*. *avenae*

The survival, reproduction, and proportion of winged offspring in the *Regiella*-infected, *Regiella*-free and *Regiella*-cured lines under different temperatures and population densities were observed. All the aphid lines were reared for more than 12 generations at 20 °C on wheat seedlings and then reared under a constant density of 30 aphids per 30 wheat seedlings in a large glass dish (20 cm diameter, 4 cm height) for three more generations before being transferred onto 7-day-old fresh wheat seedlings in a Petri dish to eliminate the possibility of parental effects. Ten wingless adults were used to produce offspring. After 24 h, all the adults were removed, and a designated number of newborn aphids (original cohort) were maintained according to the population density of 5, 10 and 15 aphids per dish. These aphids were exposed to 25, 28 and 31 °C for 4 h per day from 10:00 to 14:00 (local time), and for the rest of each day, they were kept at 20 °C. After 5–7 days, the winged or wingless aphid morphs could be clearly observed. The number of winged and wingless aphids was counted, and the proportion of the winged morphs was computed. The survival and reproduction of all the winged and wingless adults were observed daily, and all the offspring were removed after recording. The observations continued until the death of the adults. Twenty, 10 and 7 replicates were performed for the population densities of 5, 10 and 15 aphids per dish at each temperature (25, 28 and 31 °C), respectively, to ensure that a total of 100 aphids were examined at each density. The fitness of each aphid population was evaluated by the intrinsic rate of increase (*r*_*m*_), which was calculated using the formula

$${r}_{m}=(ln{R}_{0})/T,\,{\rm{where}}\,{R}_{0}=\sum {l}_{x}{m}_{x},T=(\sum x{l}_{x}{m}_{x})/(\sum {l}_{x}{m}_{x}),$$

where *l*_*x*_ is the proportion of individuals in the original cohort alive at age *x*, and *m*_*x*_ is the mean number of female progeny produced per female alive in the age interval *x*.

### Data analyses

The differences in infection frequency of facultative endosymbionts in *S*. *avenae* populations and aphid genotypes sampled from different months of the year were analysed using Chi-square tests by the cross-tabular frequency table method. The transmission rates of *Regiella* between generations were analysed using a generalized linear model with binomial errors to test the effects of temperature and genotype. The *r*_*m*_ and proportion of the winged adults of aphids were analysed using the generalized linear mode (GLM) method to check the effects of *Regiella* infection, antibiotic treatment, temperature and population density on all genotypes of aphids infected and uninfected with *Regiella*. Due to the *Regiella*-infected and uninfected aphid strains belonging to different genotypes, in the GLM analysis, the genotype of aphids was considered as a covariant factor, and the *Regiella* infection, temperature, antibiotic treatment and population density were the fixed factors. The factor of *Regiella* infection means whether aphids originally carried *Regiella* or not. The factor of antibiotic treatment means whether aphids infected and uninfected with *Regiella* were treated with antibiotics or not. The differences in the *r*_*m*_ or the proportion of winged morph in aphids with the same genotype between the antibiotic treatment and control were analysed using Student’s t test. All the data were tested for normality using the Shapiro-Wilk method. The proportional data that did not meet the normal distribution were arcsine-transformed before statistical analysis. All statistical analyses were performed with SAS 9.0 software^49^.

## Results

### Facultative endosymbionts in grain aphid populations

More than 40 percent of the grain aphids in Nanjing did not house the eight known species of endosymbionts in aphids (Fig. 1). Four species of facultative endosymbionts, *Regiella*, *Hamiltonella*, *Serratia* and *Rickettsia*, were identified, which formed ten types of infection patterns in aphid populations. Aphids co-infected with two or more species of facultative endosymbionts occurred less frequently, and only co-infection with *Rickettsia* and *Serratia* was found at frequencies of 0–20% from December to March of 2014–2016 (Fig. 1). The species composition of facultative endosymbionts in the grain aphid populations of Nanjing was significantly different between months in 2014–2015 (*x*^2^ = 276.1, *df* = 50, *P* < 0.0001, Fig. 1A) and in 2015–2016 (*x*^2^ = 199.1, *df* = 35, *P* < 0.0001, Fig. 1B). *Rickettsia* and *Serratia* were predominant from December to February, whereas *Regiella* were predominant from March to May. The infection rates of *Regiella* in the grain aphid populations increased with increasing temperature. *Hamiltonella* was found in April and May, with a lower infection rate (Fig. 1).

### Effects of aphid genotype, temperature and generation on transmission of *Regiella*

**Figure 2:**
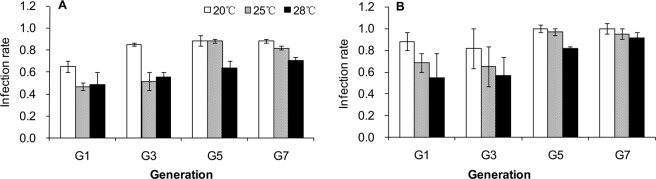
Transmission efficiency of *Regiella* from mother aphids to offspring in R1 (**A**) and R2 (**B**) aphid lines reared at 20, 25 and 28 °C for one to seven generations.

The transmission rate of the endosymbiont *Regiella* from mother to offspring was significantly affected by the aphid genotype, temperature and rearing generation (Table 1). The infection rate in aphids reared at a high temperature was lower, but the infection rate increased with the generation number for the aphids reared at high temperature. After seven generations, the infection rate increased to 70–100% at the high temperature of 28 °C. The infection rate was lower for the genotype R1 than R2 (Fig. 2).

**Table 1:** Analysis of the effects of aphid genotype, temperature and rearing generation on the transmission rate of *R*. *insecticola* using a generalized linear mode with binomial error.

Source	Maximum likelihood Chi-Square	DF	P
Genotype	48.591	1	<0.001
Temperature	68.000	2	<0.001
Generation	119.730	3	<0.001
Genotype × Temperature	7.630	2	0.022
Genotype × Generation	21.819	3	<0.001
Temperature × Generation	14.926	6	0.021
Genotype × Temperature × Generation	8.578	6	0.199

### Effect of *Regiella* on winged morph production of aphids

**Figure 3:**
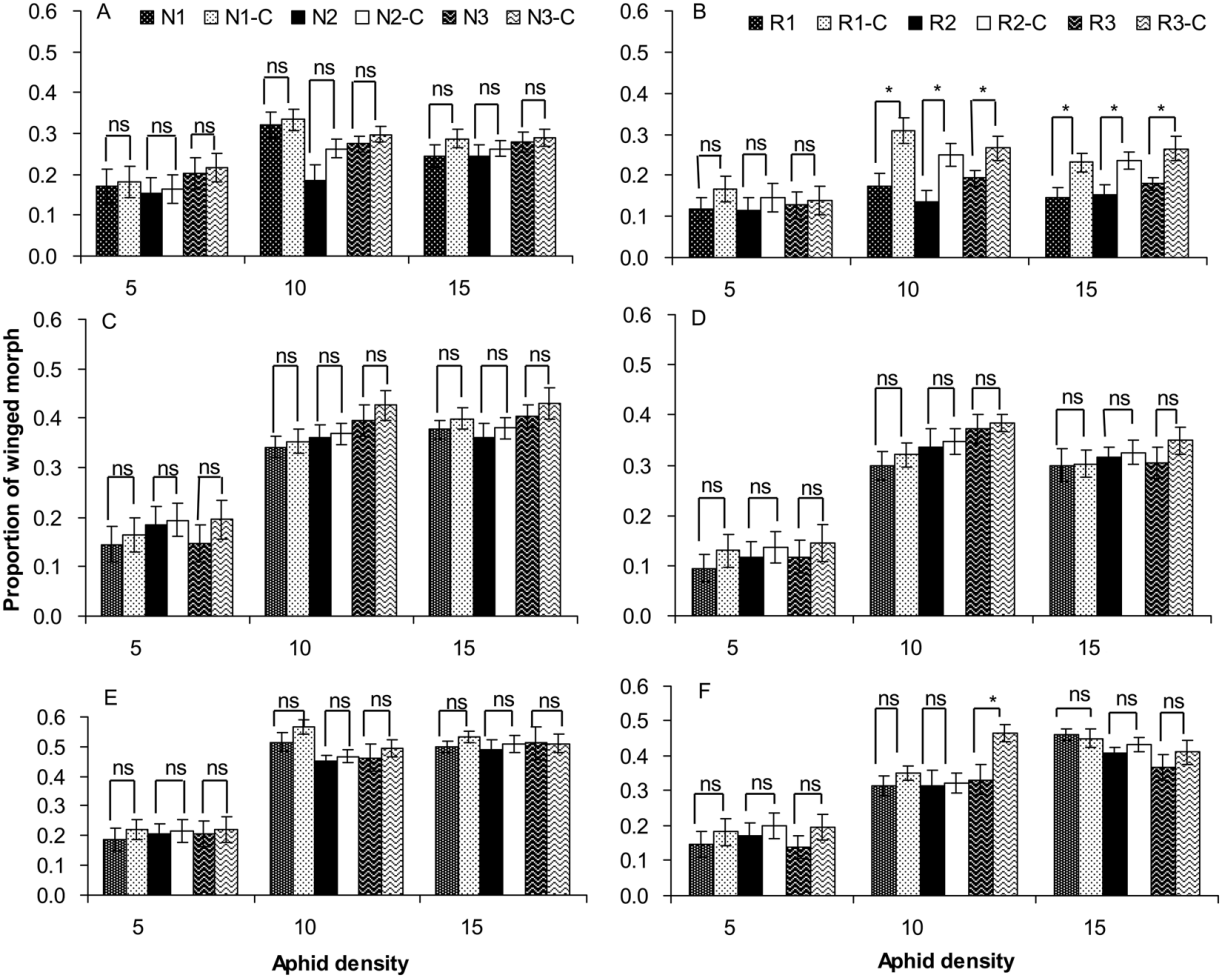
Proportion of the winged morph in the *Regiella*-free (**A**,**C**,**E**) and *Regiella*-infected (**B**,**D**,**F**) lines treated with antibiotics and exposed to 25 °C (**A**,**B**), 28 °C (**C**,**D**) and 31 °C (**E**,**F**) with different population densities. *Regiella*-infected lines: R1, R2 and R3. *Regiella*-free lines: N1, N2 and N3. Symbiont-cured lines treated with antibiotics: R1-C, R2-C, R3-C, N1-C, N2-C and N3-C. ** and * above the bars indicate significant differences between the antibiotic treatment and control at *P* = 0.01 and 0.05 levels, respectively. ns indicates no significant differences at the *P* = 0.05 level.

The production of winged offspring was significantly affected by *Regiella* infection, antibiotic treatment, temperature, and aphid density. Moreover, the interactions of *Regiella* with temperature and aphid density on aphid winged morph production were significant (Table 2). Exposed to 25, 28 and 31 °C, the *Regiella*-free aphids produced a similar number of winged offspring as those aphids treated by antibiotics under low and high population densities (Fig. 3A,C,E). Similarly, under a low population density (5 aphids per dish), *Regiella*-infected aphids also produced the same number of winged offspring as the *Regiella*-cured aphids treated by antibiotics. The antibiotic treatment did not affect the winged morph production of aphids uninfected with *Regiella*. However, exposed to 25 °C, the *Regiella*-infected aphids under a high population density (10 or 15 aphids per dish) would produce less winged offspring than these *Regiella*-cured aphids (Fig. 3B), whereas the effect of *Regiella* on winged morph production was not significant when aphids were exposed to a high temperature of 28 or 31 °C (Fig. 3D,F) with the exception of the R3 genotype at 31 °C (Fig. 3F). These results showed that the effect of *Regiella* on aphid winged morph production was dependent on environmental temperature and aphid density.

**Table 2:** GLM analysis of the effects of *Regiella* infection, antibiotic treatment, temperature and aphid density on the proportion of winged morphs in *S*. *avenae* populations.

Source	Type III Sum of Squares	DF	F	P
Aphid genotype	0.029	1	1.714	0.191
*Regiella* infection (A)	0.500	1	29.600	<0.001
Antibiotic treatment (B)	0.318	1	18.820	<0.001
Temperature (C)	3.861	2	114.266	<0.001
Density (D)	10.791	2	319.364	<0.001
A × B	0.033	1	1.979	0.160
A × C	0.074	2	2.181	0.113
A × D	0.089	2	2.634	0.072
B × C	0.039	2	1.150	0.317
B × D	0.023	2	0.676	0.509
C × D	1.691	4	25.029	<0.001
A × B × C	0.030	2	0.901	0.406
A × B × D	0.005	2	0.139	0.871
A × C × D	0.168	4	2.480	0.042
B × C × D	0.049	4	0.725	0.575
A × B × C × D	0.014	4	0.208	0.934

Aphid genotype was considered as a covariant factor. *Regiella* infection means whether the aphids were originally infected with *Regiella* or not. Antibiotic treatment means that the aphids were treated with antibiotics whether they originally carried *Regiella* or not.

### Effect of *Regiella* on fitness of aphids

**Figure 4:**
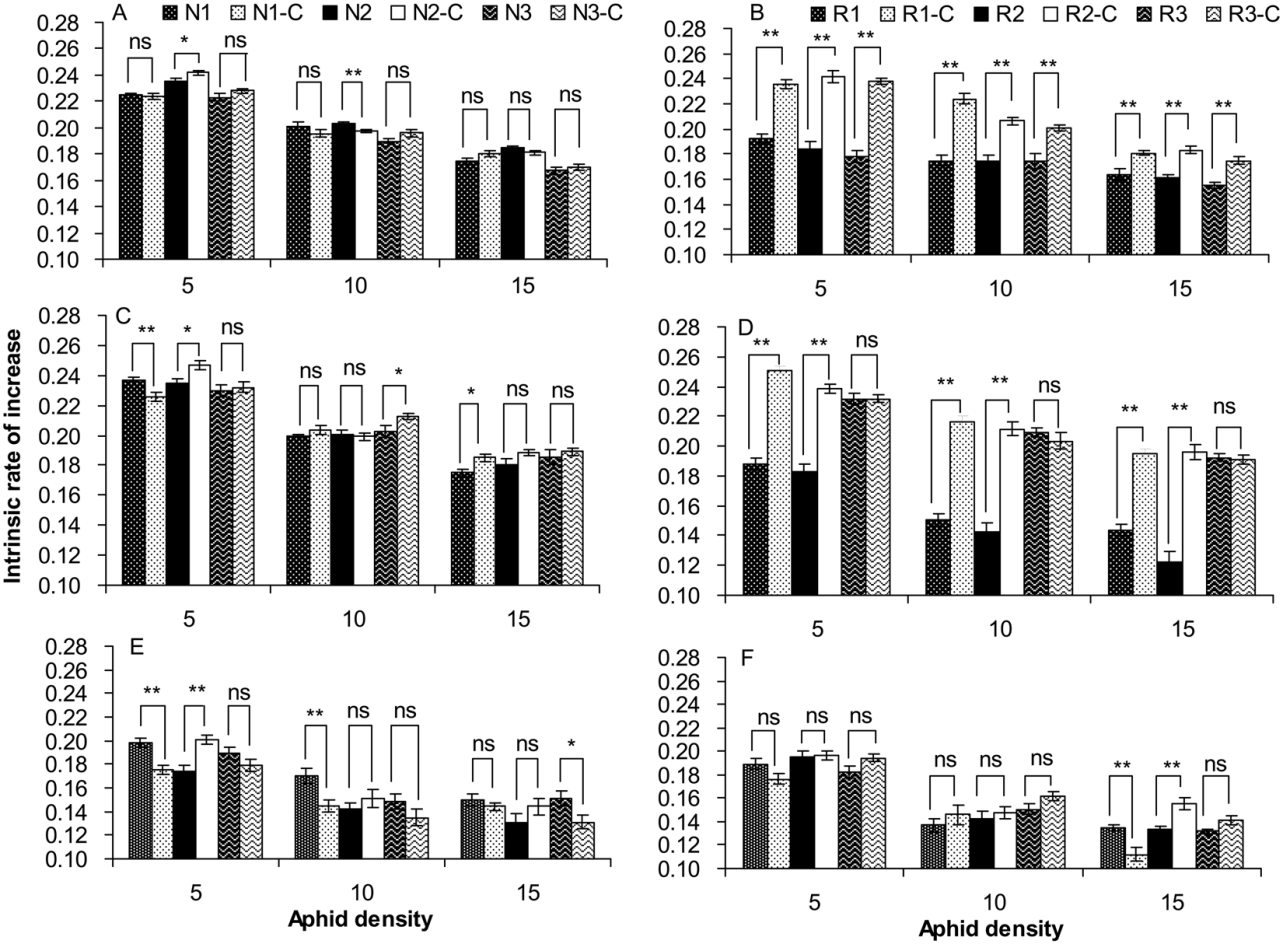
Intrinsic rate of increase of the *Regiella*-free (**A**,**C**,**E**) and *Regiella*-infected (**B**,**D**,**F**) lines treated with antibiotics and exposed to 25 (**A**,**B**), 28 (**C**,**D**) and 31 °C (**E**,**F**) with different population densities. *Regiella*-infected lines: R1, R2 and R3. *Regiella*-free lines: N1, N2 and N3. Symbiont-cured lines treated with antibiotics: R1-C, R2-C, R3-C, N1-C, N2-C and N3-C. ** and * above the bars indicate significant differences between the antibiotic treatment and control at *P* = 0.01 and 0.05 levels, respectively. ns indicates no significant differences at *P* = 0.05 level.

The *r*_*m*_ of aphids was affected by *Regiella* infection, antibiotic treatment, temperature, and aphid density. The effect of *Regiella* on the *r*_*m*_ interacted with antibiotic treatment, temperature, and density (Table 3). When exposed to 25 °C, the *r*_*m*_ values of all three *Regiella*-free lines were not significantly different from those of the adults treated by antibiotics with the exception of the line N2 at the densities of five and 10 aphids per dish (Fig. 4A). However, the *r*_*m*_ values of all three *Regiella*-infected lines were significantly lower than those of their corresponding *Regiella*-cured lines at 25 °C, regardless of the aphid density (Fig. 4B). When exposed to 28 °C, the antibiotic treatment decreased the *r*_*m*_ of the *Regiella*-free line N1 at a density of five aphids per dish and increased the *r*_*m*_ of the N1 line at a density of 15 aphids per dish, the N2 at a density of five aphids per dish, and the N3 line at a density of 10 aphids per dish (Fig. 4C). However, the *r*_*m*_ values of the *Regiella*-infected lines R1 and R2 significantly increased when *Regiella* was cured by antibiotics under all three densities, whereas the *r*_*m*_ of the R3 line was similar to that of the R3-C line (Fig. 4D). Exposure to 31 °C was harmful for grain aphids, and the *r*_*m*_ was lower overall than at 25 and 28 °C (*Regiella*-free aphids: *F*_2, 666_ = 617.02, *P* < 0.001; *Regiella*-infected aphids: *F*_2, 666_ = 288.87, *P* < 0.001). When exposed to 31 °C, the antibiotic treatment resulted in a decrease of the *r*_*m*_ in the N1 line at a density of five and 10 aphids per dish and the N3 line at a density of 15 aphids per dish (Fig. 4E). However, the antibiotic treatment did not affect the *r*_*m*_ of the *Regiella*-infected lines at densities of five and 10 aphids per dish. At a high density of 15 aphids per dish, the *r*_*m*_ of the R1 line was significantly higher than that of the R1-C line, but the *r*_*m*_ of the R2 line was significantly lower than that of the R2-C line, whereas the *r*_*m*_ of the R3 line was similar to that of the R3-C line (Fig. 4F). Overall, the effect of antibiotic treatment on the *r*_*m*_ was generally positive in the aphids carrying *Regiella*, but it was neutral in the majority of the aphids uninfected with *Regiella*; therefore, the interaction between *Regiella* infection and antibiotic treatment was significant (Fig. 4; Table 3). The effect of *Regiella* infection on aphid fitness was significantly dependent on environmental temperature, aphid density and aphid genotype.

**Table 3:** GLM analysis of the effects of *Regiella* infection, antibiotic treatment, temperature and aphid density on the intrinsic rate of increase of *S*. *avenae* populations.

Source	Type III Sum of Squares	df	F	P
Aphid genotype	0.001	1	2.833	0.093
*Regiella* infection (A)	0.009	1	27.264	<0.001
Antibiotic treatment (B)	0.051	1	151.965	<0.001
Temperature (C)	0.408	2	613.621	<0.001
Density (D)	0.541	2	814.102	<0.001
A × B	0.051	1	152.968	<0.001
A × C	0.006	2	8.540	<0.001
A × D	0.001	2	0.873	0.418
B × C	0.030	2	44.407	<0.001
B × D	0.001	2	1.700	0.183
C × D	0.008	4	6.131	<0.001
A × B × C	0.011	2	16.758	<0.001
A × B × D	0.002	2	2.444	0.087
A × C × D	0.006	4	4.152	0.002
B × C × D	0.005	4	4.053	0.003
A × B × C × D	0.004	4	2.780	0.026

Aphid genotype was considered as a covariant factor. *Regiella* infection means whether the aphids were originally infected with *Regiella* or not. Antibiotic treatment means that the aphids were treated with antibiotics whether they originally carried *Regiella* or not.

## Discussion

The infection pattern of endosymbionts in insect populations is environmentally dependent. This study found that the grain aphid populations in Nanjing hosted four species of facultative endosymbionts, *Regiella*, *Hamiltonella*, *Serratia* and *Rickettsia*, which formed ten types of infection patterns, and the infection patterns varied with season. It has been found that endosymbiont species or the dominant endosymbiont species in insect populations are different in different regions^41,42,50^. The grain aphids in Oxfordshire, South East England harboured *Regiella*, *Hamiltonella* and *Serratia*, and *Hamiltonella* and *Regiella* were the predominant endosymbionts^42^. In Germany, grain aphids were also infected with the endosymbionts *Hamiltonella* and *Regiella*^41^. Here, we found that *Rickettsia*, *Serratia* and *Regiella* were prevalent in the grain aphid populations in Nanjing, but the infection rate of *Hamiltonella* was low. The different infection patterns of facultative endosymbionts in insect populations might result from environmental factors. Temperature, host plant and precipitation are important factors that determine the distribution of *Regiella*^50^. The incidence of *Serratia* in pea aphids was higher in summer than in spring^22^. The chestnut weevil *Curculio sikkimensis* is infected at a higher frequency with *Wolbachia* and *Rickettsia* at localities with higher temperatures but at a lower frequency at localities with greater snowfall^4^. In this study, we found that the infection frequency of *Regiella* in the grain aphids collected in the spring was higher than in the aphids collected in the winter. Temperature may be a key factor in determining the temporal and spatial distribution of endosymbionts in insect populations.

Vertical transmission of endosymbionts in hosts was influenced by environmental temperature^51^. Pea aphids harbouring *Regiella* were vulnerable to high temperature^5^, so the infection frequency of *Regiella* in aphid populations might decrease under high temperature. High temperature significantly decreased the relative density of *Hamiltonella* in whiteflies^52^, which might lead to a decreased transmission rate. In this study, we found a lower transmission rate of *Regiella* in the grain aphids at high temperature; however, the endosymbiont had the potential to adapt to heat. When aphids were reared for several generations at high temperature, the transmission rate of *Regiella* increased up to 100%. The adaptation of an endosymbiont to environments in a short time improves its prevalence. The recent prevalence in eastern North America and spreading of the endosymbiont *Spiroplasma* from east to the west across the continent was found in *Drosophila neotestacea*^6^; these results showed that the infection frequency of this endosymbiont was dynamic on spatial and temporal scales and that the endosymbiont might be acclimatizing. When the endosymbiont fully adapts to the environment, the infection frequency would be expected to stabilize.

The roles of endosymbionts in insect hosts are conditional. In a previous study, *Regiella* had a negative effect on the fitness of the grain aphids^7^. In this study, the fitness cost of *Regiella* on the grain aphid was also found. However, we found that the negative effect of *Regiella* on aphid fitness was not constant but environmentally dependent. The negative effect of *Regiella* only occurred under some specific conditions, such as a specific range of temperatures and aphid densities. Exposed to 25 and 28 °C, *Regiella* infection exhibited a negative effect on the grain aphids, but when exposed to 31 °C, the effect was complex, depending on the aphid density and genotype. A previous study found that reduced fecundity of pea aphids resulting from *Rickettsia* was strongly dependent on temperature and the host plant on which aphids fed^21^. The effects of the facultative symbionts *Regiella*, *Hamiltonella* and *Serratia* on pea aphids were also dependent on when and whether the aphids were exposed to heat stress^5^. In addition, *Regiella* had different effects on the sexual induction profiles in different lineages of pea aphids^27^. The combination of the genetic background of hosts and environmental factors modulates the performance of facultative endosymbionts in insect hosts.

The interactions of endosymbionts and environmental conditions affect the population dynamics of insects and the infection pattern of endosymbionts. Shifts in the production of winged and wingless morphs are an adaptive strategy of insects to environments^53^. The aphid’s winged morph production is affected not only by environmental conditions, such as the host plant’s quality^29^ but also by the infection of endosymbionts^21,25^. We found that the grain aphids harbouring *Regiella* produced fewer alates when exposed to 25 °C than the *Regiella*-uninfected and cured aphids, but there were no significant differences when aphids were exposed to 28 and 31 °C. These results imply that the effect of an endosymbiont on winged production of aphids is dependent on environmental temperature. At relatively lower temperatures, *Regiella* inhibits the production of alates and the reproduction of aphids. Those *Regiella*-infected aphids will disperse less while the *Serratia-* and *Rickettsia*-infected aphids will produce more winged morphs for dispersal^21^. The lower population growth and less dispersal at lower temperatures may reduce the sampling or frequency of the infected individuals in the field. When temperature increases and becomes unsuitable, the aphids infected with *Serratia* and *Rickettsia* will produce winged morphs to migrate towards other areas. Therefore, the infection rates of *Regiella* in aphid populations increased as *Rickettsia* and *Serratia* decreased.

The migration and meeting of several aphid populations from different regions or host plants might lead to changes in the infection pattern of the endosymbionts. Aphids collected from different host plants might have strong genetic differentiation and be infected with different facultative symbiotic bacteria^54,55^. In this study, we found that the *Regiella*-infected and uninfected aphids belonged to different genotypes. This result suggested that the loss and acquisition of the *Regiella* endosymbiont in natural aphid populations occurred infrequently, and the endosymbiont infection pattern in the grain aphids was associated with the genetic structure of the aphid populations. Migration of aphid populations from different regions might result in the various infection patterns of endosymbionts. Because the immigration period of this aphid in Nanjing occurs in the autumn but not in the spring^38–40^, the movement of aphids carrying other symbionts, such as *Serratia*, could be one of reasons causing the changes in infection patterns of the endosymbionts during winter and spring.

Although the transmission of *Regiella* improved over time if a high temperature environment was experienced, *Regiella* was costly or neutral at all temperatures. This suggests that it is unlikely to be selection that leads to the increase of *Regiella* in field populations of aphids. In this study, we did not examine the role of *Regiella* in improving the protection of aphids against the fungus *Pandora*^11,56^. Entomogenous fungi are more prevalent in aphid populations in the spring than in the winter. The aphids hosting a protective endosymbiont against fungus may flourish in the warm season. Therefore, the infection pattern of endosymbionts in the grain aphid populations shifted from *Rickettsia* and *Serratia* in the winter to *Regiella* in the spring.

Antibiotic treatment and microinjection with body fluids are common methods to artificially manipulate (remove and acquire) endosymbionts in insects, and the roles of many endosymbionts have been determined using these methods^5,31–33^. In this study, we treated both the *Regiella*-infected and uninfected aphids with antibiotics. We found that the antibiotic treatment did not alter the winged morph production of *Regiella*-uninfected aphids but increased the production rate of winged-aphids in *Regiella*-infected aphids. This result shows that the antibiotic treatment of the *Regiella*-infected aphids is sufficient for disentangling the effect of this endosymbiont on the winged morph production of the grain aphids. However, the antibiotic treatment affected the *r*_*m*_ not only of the *Regiella*-infected aphids but also a portion of the *Regiella*-uninfected aphids under a certain condition. Of course, the effect size of the antibiotic treatment compared to the effect of the symbiont was small. Therefore, antibiotic treatments for both the infected and uninfected aphids are necessary to assess the effects of the endosymbiont on host fitness. Similarly, the inter-microinjection of body fluids between infected and uninfected insects would be better than the unidirectional injection of body fluids from the infected insects to the uninfected ones when exploring the roles of an endosymbiont in hosts.

In sum, the infection frequency and negative effect of an endosymbiont on insect hosts were dependent on environmental conditions. The facultative endosymbionts are beneficial or costly for their hosts depending on the environmental conditions.

### Ethical approval

This article does not contain any studies with human participants performed by any of the authors.

## Supplementary information

Supplementary table S1 and S2

## Data Availability

All data generated or analysed during this study are included in this published article and its Supplementary Information files.
