# Supplementary material for: Infection pattern and negative effects of a facultative endosymbiont on its insect host are environment-dependent
Source: Sci Rep. 2019 Mar 8;9:4013. doi: 10.1038/s41598-019-40607-5 (PMC6408509; doi:10.1038/s41598-019-40607-5)
Supplement: Supplementary file 1 — Supplementary table S1 and S2 [file 41598_2019_40607_MOESM1_ESM.pdf]

Infection pattern and negative effects of a facultative endosymbiont on its insect host are environment-dependent

Xiang-Dong Liu*, Hai-Xia Lei, & Fang-Fang Chen

Department of Entomology, Nanjing Agricultural University, Nanjing 210095, China

*Corresponding author: E-mail: liuxd@njau.edu.cn. Tel:+0086-25-8439 5242

Table S1. Primer sequence for PCR detection of endosymbionts in *Sitobion avenae*

Symbiont	Primer	Primer sequences (5'-3')	Annealing temperature	Reference
Buchnera	B-16SF	GAGCTTGCTCTCTTTGTCGGCAA	55 °C	Tsuchida *et al*., 2002
aphidicola	B-161R	CTTCTGCGGGTAACGTCACGAA		
Regiella	10F	AGTTTGATCATGGCTCAGATTG	55 °C	Sandström *et al*., 2001
insecticola	R-U433	GGTAACGTCAATCGATAAGCA		Ferrari *et al*., 2012
Hamiltonella	10F	TACGGYTACCTTGTTACGACTT	55 °C	Sandström *et al*., 2001
defensa	H-T419	AGTGAGCGCAGTTTACTGAG		Ferrari *et al*., 2012
Serratia	10F	AGTTTGATCATGGCTCAGATTG	55 °C	Sandström *et al*., 2001
symbiotic	S-R433	CTTCTGCGAGTAACGTCAATG		Ferrari *et al*., 2012
Rickettsia	16SA1	AGAGTTTGATCMTGGCTCAG	55 °C	Fukatsu and Nikoh, 1998
	16SA2	TCCACGTCACCGTCTTGC		Sakurai *et al*., 2005
Rickettsiella	RCL16S-211F	GGGCCTTGCGCTCTAGGT	55 °C	Tsuchida *et al*., 2010
	RCL16S-470R	TGGGTACCGTCACAGTAATCGA		
Spiroplasama	16SA1	AGAGTTTGATCMTGGCTCAG	54 °C	Fukatsu and Nikoh, 1998
	Spi16SR	ATCATCAACCCTGCCTTTGG		McLean *et al*., 2011
Wolbachia	WSP-81F	TGGTCCAATAAGTGATGAAGAAAC	53 °C	Zhou *et al*., 1998
	WSP-691R	AAA AATTAAACGCTACTCCA		
X-type	10F	AGTTTGATCATGGCTCAGATTG	55 °C	Sandström *et al*., 2001
	X-420	GCAACACTCTTTGCATTGCT		Ferrari *et al*., 2012

Table S2. Aphid genotypes and strains used in this study

Aphid line	Genotype	*Regiella*	Allelic combination at microsatellite locus					
			S49	Sm10	Sm12	Sav1	Sav2	Sav4
R1	I	Infected	141,159	160,178	153,171	120,140	185,188	163,169
R2	II	Infected	147,153	160,178	153,171	120,140	185,188	162,176
R3	III	Infected	91,97	160,178	153,171	143,159	185,188	162,176
N1	IV	Free	97,106	160,172	158,163	122,130	183,190	158,163
N2	V	Free	110,136	160,172	155,176	120,140	185,190	158,163
N3	VI	Free	120,127	158,162	155,176	120,140	185,190	158,163
R1-C	I	Cured	141,159	160,178	153,171	120,140	185,188	163,169
R2-C	II	Cured	147,153	160,178	153,171	120,140	185,188	162,176
R3-C	III	Cured	91,97	160,178	153,171	143,159	185,188	162,176
N1-C	IV	Cured	97,106	160,172	158,163	122,130	183,190	158,163
N2-C	V	Cured	110,136	160,172	155,176	120,140	185,190	158,163
N3-C	VI	Cured	120,127	158,162	155,176	120,140	185,190	158,163

Reference

1. Ferrari J, West J A, Via S, Godfray H C J. Population genetic structure and secondary symbionts in host-associated populations of the pea aphid complex. Evolution, 2012, 62(2):375-390.
2. Fukatsu T, Nikoh N. Two intracellular symbiotic bacteria from the mulberry psyllid *Anomoneura mori* (Insecta, Homoptera). Applied and Environmental Microbiology, 1998,64(10):3599-3606.
3. McLean M, Ferrari A J, Godfray H C J. Effects of bacterial secondary symbionts on host plant use in pea aphids. Proceedings of the Royal Society B: Biological Sciences, 2011, 278(1706): 760-766.
4. Sakurai M, Koga R, Tsuchida T, Meng X Y, Fukatsu T. *Rickettsia* symbiont in the pea aphid *Acyrthosiphon pisum*: Novel cellular tropism, effect on host fitness, and interaction with the essential symbiont *Buchnera*. Applied and Environmental Microbiology, 2005, 71(7): 4069-4075.

5. Sandström J P, Russell J A, White J P. Independent origins and horizontal transfer of bacterial symbionts of aphids. Molecular Ecology, 2001, 10(1): 217-228.
6. Tsuchida T, Koga R, Shibao H. Diversity and geographic distribution of secondary endosymbiotic bacteria in natural populations of the pea aphid, *Acyrthosiphon pisum*. Molecular Ecology, 2002, 11(10):2123-2135.
7. Tsuchida T, Koga R, Horikawa M. Symbiotic bacterium modifies aphid body color. Science, 2010, 330(6007): 1102-1104.
8. Zhou W, Rousset F, O'Neil S. Phylogeny and PCR-based classification of *Wolbachia* strains using *wsp* gene sequences. Proceedings of the Royal Society B: Biological Sciences, 1998, 265(1395):509-515.
